# Dr. A. K. Gurwara

**Published:** 2010

**Figure:**
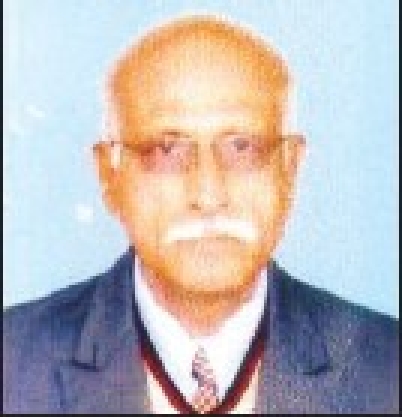
22^nd^ September 1949 – 10^th^ February 2010

With profound grief, ISA, Jhansi city branch of UP chapter, informs the sad demise of Dr. A. K. Gurwara, Rtd. Professor and Head, Department of Anaesthesia, M.L.B. Medical College, Jhansi, on 10^th^ February 2010. He was born on 22^nd^ September 1949. Dr. Gurwara passed his MBBS, DA and MS (Anaesth.) from M.L.N. Medical College, Allahabad, and joined M.L.B. Medical College, Jhansi, in 1976 as a lecturer. Thenceforth, on being promoted, he went on to become the head of the department and served as the head since July 2000 and retired from active service on 30^th^ September 2009. He is survived by his wife, a son and a daughter. He was an active life member of ISA and was instrumental in the creation of Jhansi city branch being its first secretary and then president. He remained a patron of the branch till his end. He organised the annual conference of UP chapter ISA in 1987 and had been its Vice President twice.

